# Nurses’ perceptions of fall risk factors and fall prevention strategies in acute care settings in Saudi Arabia

**DOI:** 10.1002/nop2.1182

**Published:** 2022-01-30

**Authors:** Adnan M. Innab

**Affiliations:** ^1^ 37850 Nursing Administration and Education Department College of Nursing King Saud University Riyadh Kingdom of Saudi Arabia

**Keywords:** Falls, nurses, nursing, patient safety, preventable falls

## Abstract

**Aim:**

This study aimed to explore nurses’ perceptions of the factors associated with falls and of fall prevention strategies in acute care settings in Saudi Arabia.

**Design:**

This is a cross‐sectional, correlational, descriptive study.

**Methods:**

Data were collected from 102 nurses using a survey on the risk factors and prevention strategies of injurious falls.

**Results:**

We found that impaired balance and muscle strength, limited mobility, and an inability to follow safety instructions were reported as the top factors in falls. Multidisciplinary fall prevention strategies are effective in reducing the prevalence of falls. Nurses with higher levels of education had higher perceptions towards the risk factors of falls. Formal patient safety training, including fall prevention education programmes, were shown to reduce falls by making nurses more aware of fall risk factors and prevention strategies. The findings of this study can be used to inform managers about factors that may contribute to falls in acute care settings.

## 1 INTRODUCTION

Falls represent a significant threat to patient safety in clinical settings and can lead to severe complications and death. The World Health Organization (WHO) estimates that around 646,000 fatal falls occur every year, making falls the second leading cause of death worldwide, has also estimated that 37.3 million falls are severe enough to require medical care (WHO, 2018).

Falls have been identified as a priority issue among hospitalized patients and can result in a prolonged hospital stay as well as increased healthcare costs (Alderby et al., 2017) and so a considerable body of literature exists on fall risk factors and prevention. Researchers have found that inpatient fall rates range from 25% to 45% (Lohse et al., 2012), and that 25% of inpatient fall injuries can be prevented (Tzeng & Yin, 2013). However, preventing inpatient falls in acute care settings remains a major challenge for healthcare providers. Researchers have suggested that in order to tailor appropriate interventions and reduce the burden of falls, preventable risk factors for falls should be comprehensively studied (Almegbel et al., 2018; Tzeng & Yin, 2013; Wilson et al., 2016).

As the factors that contribute to falls are multidimensional, risk factors are classified into intrinsic and extrinsic factors (Alderby et al., 2017; Hill et al., 2018; Tzeng & Yin, 2013). Intrinsic risk factors are those relating to the patient themselves, including age, history of falls, poor balance and walking ability, weakness in the lower extremities, history of Parkinson's disease, dizziness and impaired vision (Hill et al., 2018; Tzeng & Yin, 2013). A number of researchers have found that patient age, muscle weakness, cognitive impairment and history of falls significantly impact the likelihood of future falls (Ahmadiahangar et al., 2018; Van Ancum et al., 2018; Deandrea et al., 2010).

Extrinsic factors are environmental conditions that affect patient safety. The use of medications, such as antidepressants, antipsychotics and sedatives, is the most commonly reported extrinsic factor in falls in Saudi Arabia (SA) (Alderby et al., 2017). Other extrinsic factors that have been identified in other countries include slippery or uneven surfaces, poor lighting, or high‐risk activities (Hill et al., 2018; Tzeng & Yin, 2013).

The purpose of this study was to explore nurses’ perceptions of the factors associated with falls and fall prevention in acute care settings in SA. The specific aims of this study were to (1) determine the frequency and effectiveness of using preventive measures for falls in inpatient settings, and (2) determine the relationship between nurses’ characteristics (age, gender, level of education, length of experience, position and recent fall prevention education) and their perceptions of why falls occur in acute care settings.

## 2 BACKGROUND

The list of anticipated risk factors for falls has typically been extracted from medical records or otherwise compiled from patients’ perspectives (Almegbel et al., 2018; Alshammari et al., 2018). Other researchers have relied on incident reports to identify and describe fall events (Castellini et al., 2017; Walsh et al., 2018), but these data are mostly lacking in essential details. For example, nurses’ knowledge of the risk factors for falls, as well as of interventions to reduce falls, is rarely documented in the literature. There is therefore a need to highlight the risk factors and interventions from the perspective of nurses. As front‐line practitioners with around‐the‐clock patient contact, nurses can give valuable information about the healthcare environment, including assessments of patient fall risks and responses.

Wilson et al. (2016) conducted a qualitative study to explore the facilitators and barriers of implementing evidence‐based fall prevention intervention based on nurses’ perceptions. Nurses identified a number of strategies that were effective in reducing the prevalence of falls, such as medication review and modification, staff education, inter‐professional collaboration and post‐fall huddles (Wilson et al., 2016). In other studies, researchers stated that instead of using generic fall risk‐reduction strategies, healthcare professionals should use fall risk scores as guidance to reduce falls in acute care settings (Chidume, 2021; Titler et al., 2016).

Numerous interventional studies have been conducted on fall prevention in acute care settings, yet the quality of reporting is inconsistent and lacks thorough description. A number of researchers evaluated the effectiveness of education on fall prevention as a single intervention (Heng et al., 2020; Lee et al., 2014) or as a part of a multifactorial interventions (Cameron et al., 2018). Educating healthcare personnel on fall prevention has been identified as a priority for improving patient safety in acute care settings (Hang et al., 2016). There is still a need for a more focused study of the influence of education programmes on fall prevention, employing behavioural change models or theoretical frameworks (Shaw et al., 2020).

In a recent review, researchers evaluated the level of evidence extracted from randomized controlled trials on fall prevention interventions in hospitals (e.g. chair and bed sensor alarms, the effect of physiotherapy in rehabilitation wards and multifactorial interventions). The authors concluded that these interventions were inconsistently effective (Cameron et al., 2018). The inconsistency between the results of the previous studies could imply that hospitals have various strengths and limitations in the care they deliver to patients, and that when the data are collected, the differences in demographic characteristics specific to each study may be concealed.

In SA, the 1‐year prevalence of falls among older adults in the capital city of Riyadh for 2018 was around 50%, of whom 74% sustained post‐fall injuries (Almegbel et al., 2018). In medical and surgical units in a teaching hospital in SA, researchers reported that only 2.4% of patient falls in a 3‐month period were reported (Alderby et al., 2017). This may indicate that the incidence of falls in acute care settings remains under‐reported. In this study, the primary investigator aimed to assess nurses’ perceptions of the factors associated with falls in acute care settings. The number of expatriate nurses in SA is increasing, but the literature lacks evidence about the risk factors for falls as reported by these expatriate nurses. To date, no study has focused specifically on the knowledge of expatriate nurses in SA.

This study was guided by the Swiss cheese model (Reason, 2000), which has been extensively used as a theoretical framework in patient safety studies (Innab, 2019; Seshia et al., 2018). The basic premise of this model is that people are fallible and incidents are expected even in the best organizations (Reason, 2000). This model equates incidents to multiple slices of Swiss cheese. Holes in the slices may occur due to intrinsic factors (e.g. unsafe acts by nurses) or extrinsic factors (e.g. having unclear policies or procedures to prevent incidents from happening), but it is not always clear which is the cause or how they relate to each other (Reason, 2000).

## 3 METHODS

This study adhered to the STrengthening the Reporting of OBservational studies in Epidemiology (STROBE) initiative, as detailed in the Supplementary File.

### 3.1 Study design

This was a cross‐sectional, correlational, descriptive study. This design was chosen because it remains unclear which of the risk factors for falls are related to the occurrence of falls specifically in acute care settings in SA hospitals. It would not be appropriate to start with a robust design, such as RCT, without understanding the risk factors for falls in SA overall.

### 3.2 Sample and study settings

This study was conducted at a teaching hospital, chosen for its mix of local and expatriate nurses and role in the medical education system. The researcher used a convenience sampling method to collect data from nurses working in acute care departments. Included nurses (a) were currently working in acute care units, (b) held a current Registered Nurse (RN) license, (c) were working as a staff or head nurse and (d) were proficient in English. Nursing students, nursing interns and nurses working in outpatient settings were excluded due to a lack of extensive work with patients at a higher risk of falls.

### 3.3 Data collection procedure

The PI met with nurses during the nursing endorsement and described the purpose of the study, potential risks and benefits, confidentiality and the privacy of participants. The PI distributed the recruitment statement and surveys after the meeting and asked the nurses to fill out the questionnaire if they were willing to participate in this study. No participant names or identifiers were collected. Participants were asked to place their survey responses in sealed envelopes to ensure confidentiality and return the surveys to the PI upon completion.

### 3.4 Measurement

The demographic form used by Tzeng and Yin (2013) was edited to reflect nursing practice in SA and was used with the authors’ approval. Demographic data collected with the form include age, gender, level of education, length of experience, position (head vs. staff nurse), recent fall prevention education (<12 months, >12 months, no training), type of unit (medical, surgical, medical‐surgical combined, ICU) and the acuity level of the unit (critical, non‐critical, other).

The dependent variables (risk factors in falls and fall prevention strategies) were measured using the survey of injurious fall risk factors and fall prevention interventions developed by Tzeng and Yin (2013). This self‐report survey consists of two general content areas: risk factors in falls (81 items) and interventions for preventing injurious falls (75 items). Participants indicated their degree of agreement with each item using a Likert‐type scale, which ranges from 1 = rarely to 5 = always (Tzeng & Yin, 2013). This tool has been previously tested in SA and has demonstrated Cronbach's alpha of 0.9 for the two content areas (Alderby et al., 2017). The time needed to fill out the questionnaire was between 15 and 25 min.

### 3.5 Ethical considerations

IRB approval was obtained prior to collecting data and permission to use the instrument was granted by the original authors (Tzeng & Yin, 2013). The PI informed the nurses that their participation in the research would be entirely voluntary and that they had the right to withdraw at any time while filling out the survey. There were no penalties for declining to participate in this study. All nurses’ responses were anonymous. Nurses were informed that the data would be reported in aggregate form to ensure that responses from specific departments would not be identifiable. Access to research files was restricted to the PI.

### 3.6 Data analysis

The optimal sample size to obtain statistical power was determined using G*Power (Faul et al., 2007). For a significance level of 0.05, a power of 0.80, an effect size of 0.15 and four predictors, a minimum sample of 85 participants is needed to perform regression analysis. Because this sample included 102 respondents, it can be assumed that these conditions were met. Data were analysed using version 26.0 of the IBM SPSS statistical program. The data analysis involved evaluating the psychometric properties of the instrument and using frequency and descriptive statistics, a *t* test and multiple linear regression. The PI used frequency and descriptive statistics to describe participants' characteristics. The descriptive statistics in this study include mean (M), standard deviation (*SD*) and range. Additionally, all assumptions of parametric tests were tested (e.g. normality, homogeneity and linearity). The data were approximately normally distributed, as assessed by histograms. Among the *t* test models, the assumption of homogeneity was violated, as assessed by Levene's test for equality of variances (*p* <.05). Therefore, the results of Welch *t* test (equal variances not assumed) were reported. Among the regression models, the scatterplots determined that the linearity and homoscedasticity assumptions were met. There were not any highly influential points, as assessed by visual inspection of the studentized deleted residuals and histogram.

## 4 RESULTS

A total of 150 hard copies of questionnaires were distributed to the eligible participants. 102 questionnaires were returned (response rate = 68%). Table 1 displays the demographic characteristics of participants. Participants’ ages ranged from 26 to 56 years, with a mean age of 38.2 (±7.9). The majority of participants (80.4%) were female. The educational level of participants ranged from college diploma to master's degree, with the majority of nurses (59.8%) holding a BSN degree.

**TABLE 1:** Demographic participants’ characteristics

Variable (range)	*n* (%) or *M* (±*SD*)
Age (26–56 years)	38.2 (±7.9)
Gender	
Male	20 (19.6)
Female	82 (80.4)
Level of education	
Diploma	33 (32.4)
BSN	61 (59.8)
MSN	4 (3.9)
Length of experience (2–32 years)	11.4 (±6.3)
Position
Staff Nurse	83 (81.4)
Head/charge nurse	13 (12.7)
Other	6 (5.9)
Recent fall prevention education
Less than 12 months ago	88 (86.3)
More than 12 months ago	3 (2.9)
No training/cannot recall	9 (8.8)
Type of unit
Medical	44 (43.1)
Surgical	13 (12.7)
Medical surgical combined	36 (35.3)
ICU	5 (4.9)
Acuity level of unit
Critical care	20 (19.6)
Non‐critical	67 (65.7)
Other	13 (12.7)

Abbreviations: BSN, Bachelor of Science in Nursing; MSN, Master of. Science in Nursing; ICU, intensive care unit.

Length of experience ranged from 2 to 32 years, with a mean experience of 11.4 (*SD* ± 6.3) years. More than two‐thirds of participants (81.4%) were working as staff nurses. Most of them worked in medical (43.1%), surgical (12.7%) or medical surgical departments (35.3%). Around two‐thirds of participants (65.7%) were working in non‐critical departments. Finally, the majority of participating nurses (86.3%) had attended a fall prevention education programme in the past 12 months.

Table 2 shows the top 10 risk factors for falls as perceived by nurses. The risk factors were divided into intrinsic and extrinsic factors. Mean Likert values ranged from 2.95 to 3.26. About intrinsic factors, the majority of nurses pinpointed the risks of a patient having impaired balance, limited mobility, impaired muscle strength, an inability to follow safety instructions, vertigo, dizziness, cognitive impairment, visual impairment or being 85 or older. About extrinsic factors, participants agreed that slippery and wet floor surfaces are among the top factors in falls.

**TABLE 2:** Top 10 risk factors and effective interventions for falls as perceived by nurses

Patient‐related risk factors	*M* ± *SD*	Ranking order
Top 10 of effective interventions to prevent falls in acute care settings		
Impaired balance	3.26 ± 1.2	1
Altered or limited mobility/gait problems	3.22 ± 1.2	2
Impaired muscle strength	3.19 ± 1.18	3
Inability to follow safety instructions	3.18 ± 1.32	4
Vertigo or complaint of dizziness	3.17 ± 1.23	5
Cognitive impairment: disorientation	3.05 ± 1.13	6
Cognitive impairment: confusion	3.02 ± 1.20	7
Slippery or wet floor surfaces	2.99 ± 1.43	8
Age 85 or older	2.96 ± 1.43	9
Visual impairment	2.95 ± 1.36	10
Top 10 of effective interventions to prevent falls in acute care settings		
Maintain call light in reach	4.54 ± 1.0	1
Collaborate with physicians	4.52 ± 0.96	2
Standardize patient education (including visual tools)	4.50 ± 0.91	3
Conduct a mental status assessment daily	4.48 ± 0.94	4
Conduct safety/post‐fall debriefing	4.47 ± 1.0	5
Keep floor surfaces clean and dry	4.47 ± 1.0	6
Toileting regimen (offer assistance)	4.46 ± 1.0	7
Complete a fall risk assessment at admission/daily	4.45 ± 1.0	8
Provide supportive chairs with armrests	4.45 ± 1.0	9
Apply fall risk identification and wrist band	4.44 ± 1.0	10

Table 2 illustrates the 10 interventions that nurses believe are most effective for preventing injurious falls. These interventions include maintaining a call light in reach, identifying the patient's fall risk by collaborating with physicians, providing education to patients at high risk of falls, assessing patients’ mental status, conducting post‐fall debriefings and keeping floor surfaces dry. Nurses also agreed on the importance of offering assistance to patients with toileting regimens, conducting fall risk assessments during admission and providing supportive chairs and wrist bands.

### 4.1 Association between a unit's level of acuity and risk factors for falls and fall prevention strategies

The independent sample *t* test was used to determine the differences between nurses’ perceptions between units with different levels of acuity (Table 3). There was no statistically significant difference in nurses’ perceptions based on the unit's level of acuity (*p* = .07), indicating that all participants agreed on the risk factors of falls in acute care settings. However, there was a statistically significant difference in mean effective intervention scores between the two groups (*p* < .01). Those working in non‐critical units (*M* = 4.58 ±0.50) were more aware of effective interventions than their counterparts in critical units [(*M* = 4.16 ± 0.96), (95% CI, 0.162 to 0.79), *t* (85) = 3.00, *p* < .01].

**TABLE 3:** Comparison of differences between nurses working in critical and non‐critical units

Dependent variables	Non‐critical Units *M* ±*SD*	Critical units *M* ±*SD*	*t*‐value	*p*‐value
Perceived risk factors of falls	2.47 ± 0.89	2.76 ± 0.52	1.85	0.07
Perceived effective interventions	4.58 ± 0.50	4.10 ± 0.96	3.00	0.000**

** *p* <.01

### 4.2 Participants’ characteristics and the risk of falls and fall intervention strategies

Multiple linear regression was used to determine the influence of nurses’ characteristics (age, gender, level of education and recent fall prevention education) on their perceptions of risk factors of falls (Table 4). Level of education was the only significant predictor; those with a higher level of education were more likely to perceive the risk factors for falls (*β* = 0.315, *t* = 2.17, *r* ^†^ = 0.236, *R* ^2^ = 0.056, *p* < .05). About intervention strategies (Table 4), nurses who had attended a fall prevention education programme in the last 12 months were more likely to be aware of effective interventions to prevent falls (*β* = −0.364, *t* = 2.69, *r* ^†^ = 0.290, *R* ^2^ = 0.084, p < .01).

**TABLE 4:** Participants’ characteristics and their perceptions towards risk of falls and effective interventions

Independent variables	*β*‐coefficients	Standardized *β*	*t*	*p*
Relationship between participants’ characteristics and their perceptions towards risk of falls				
Age	0.004	0.043	0.390	0.697
Gender	0.176	0.089	0.796	0.428
Level of education*	0.315	0.238	2.175	0.032
Fall prevention education	0.146	0.113	1.009	0.316
Model Summary	*r* ^†^ = 0.236	*R* ^2^ = 0.056		0.278
Participants’ characteristics and their perceptions towards effective interventions to prevent falls				
Age	−0.012	−1.125	−1.160	0.249
Gender	−0.015	−0.008	−0.075	0.941
Level of education	−0.103	−0.082	−0.758	0.451
Fall prevention education**	−0.364	−0.296	−2.69	0.009
Model Summary	*r* ^†^ = 0.290	*R* ^2^ = 0.084		0.09

^a^ Independent variable in the regression model.

* Significant variable (*p* < 0.05);

** Significant variable (*p* < 0.01).

## 5 DISCUSSION

This study aimed to explore nurses’ perceptions of the factors associated with falls and fall prevention strategies in acute care settings in Saudi Arabia. The participants in this study indicated that all newly recruited nurses are required to attend an educational programme that discusses critical topics such as patient safety. During that programme, nurses are encouraged to use a fall risk assessment tool for all admitted patients to identify patients at risk of falls.

In this study, we found that nurses mostly agree about the intrinsic and extrinsic factors contributing to falls in acute care settings. This result is consistent with previous research (Alderby et al., 2017; Tzeng & Yin, 2013) demonstrating that intrinsic factors (e.g. impaired balance, muscle strength, vision and cognition, as well as mobility problems, vertigo, dizziness and failure to follow safety rules) are among the top risk factors in falls. Being 85 or older was another patient factor highlighted by the nurses in this study. Deandrea et al. (2010) conducted a systematic review and found that intrinsic factors (e.g. age, muscle weakness and gait and balance disorders) were among the top risk factors for falls in community‐dwelling older people. However, the measurements used in these two studies were not comparable, and the researchers did not generate a summary estimate (Deandrea et al., 2010). Although our finding is not consistent with previous studies, this could reflect the age groups of patients who are at risk of falls in SA. About extrinsic factors, researchers had previously found that slippery and wet floor surfaces were the most frequent cause of falls in acute care settings (Alderby et al., 2017; Lee et al., 2018; Tzeng & Yin, 2013).

A number of researchers have found that certain types of medications (e.g. hypnotics, sedatives and antipsychotics) may cause impaired balance and coordination, thereby increasing the risk of falls (Aryee et al., 2017; Li et al., 2018; Sterke et al., 2012). In a retrospective case‐control study, the authors found a significant association between the use of psychotropic agents and injurious falls (Aryee et al., 2017). Other researchers have concluded that antihistamine and anticholinergic drugs may impact cognitive abilities and lead to blurred vision, which in turn can enhance the risk of falls (Prevention & Panel, 2001). Additionally, in a study by Shuto et al. (2010), the researchers collected data from incident reports between 2003 and 2005 and found that the use of antihypertensive, antiparkinsonian and anti‐anxiety medications was significantly associated with an increased risk of falls in hospitals. All in all, patients who are using multiple medicines that increase the risk of falls need to be monitored more closely. Pharmacists could play a significant role in modifying the type or dose of the medicine‐related fall risk (Akande‐Sholabi et al., 2020).

Although the majority of nurses in this study are expatriate nurses, who came from different countries and had different backgrounds, they did not perceive the use of various medications as being related to the risk of falls in acute care settings. This could be due to the restrictions on medication use in the teaching hospital that are enforced by nurses. For instance, those at a high risk of falling could be identified prior to administering medications to avoid further increasing their risk.

Teamwork between nurses and other healthcare providers should be the top priority in improving fall prevention. In this study, nurses agreed about the importance of a number of interventions to prevent or reduce falls in acute care settings. These interventions can be best described as multidisciplinary fall prevention strategies (Alderby et al., 2017). Only two of the interventions were related to the physical environment of patients’ rooms: maintaining a call light in reach and keeping floor surfaces dry. A previous study (Tzeng & Yin, 2013) found that environmental interventions often focused on an unsafe care environment.

The remainder of the interventions endorsed by the nurses in this study include identifying the patient's fall risk by collaborating with physicians, educating hospital patients to prevent falls, assessing patients’ mental status, conducting post‐fall debriefings, conducting a fall risk assessment during admission, assistance patients with their toileting regimens and providing supportive chairs and wrist bands. Previous studies have also found that these interventions are frequently used and effective for fall prevention (Alderby et al., 2017; Tzeng & Yin, 2013), although the nurses in these studies reported that locking hospital bed brakes, keeping patients’ beds in low positions and orienting patients to their physical environment were the most effective interventions of all.

The results of this study showed that participants working in general wards had higher perceptions than those working in critical care units. This may relate to the health status of patients, as those in critical care units are less mobile (more likely to be bedridden) than patients in non‐critical care units. Based on the guidelines of the Saudi Commission for Health Specialties (2015), the ratio of nurses to patients in ICUs must be 1:1 or 1:1.5. Maintaining this ratio can help nurses observe patients closely throughout the shift. Decreasing the ratio of patients to nurses could significantly enhance the level of patient care and thus reduce the prevalence of falls in acute care.

We also found that participants with a higher level of education were more attuned towards the risk factors for falls. Previous studies have not found any significant difference in nurses’ perceptions towards the risk of falls according to their level of education (Alderby et al., 2017). The dearth of research on the nurses’ characteristics and risk factors for falls constituted a roadblock for making effective comparisons. Furthermore, nurses who attended training courses in the last 12 months were more likely to perceive the suggested interventions as being effective for fall prevention. This result is consistent with previous research (Alderby et al., 2017; El Enein et al., 2012; Kim & Seo, 2017), which indicated that attending training courses on patient safety enhanced nurses’ knowledge and performance about fall prevention strategies.

### 5.1 Limitations and recommendations for future research

This study has certain limitations. First, the data were collected at a single point in time from a sample of nurses working in a single teaching hospital in SA. Second, the researcher used a convenience sampling method, reducing the generalizability of the findings to other healthcare settings. Future researchers are recommended to use a robust design and sampling method to enhance the external validity of the study.

Other healthcare systems may have different fall prevention protocols; thus, the emphasis on fall prevention strategies in this hospital may reduce the generalizability of the findings to other settings. There is also a need for feasible interventions that prevent inpatient falls. Testing the effectiveness of educational programmes on fall prevention strategies with regards to nurses’ knowledge and skills is also warranted. It is also recommended to study the link between an institution's fall prevention strategies and the prevalence of falls.

### 5.2 Implications for nursing practice

The findings of this study can be used to inform managers about factors that may contribute to falls in acute care settings. The findings of this study have also indicated that a routine and formal patient safety training once a year is an effective approach to identify effective fall practices and thus reduce the prevalence of falls. Adequate number of nursing staff in a unit can be directly linked to the fall rates in the hospital.

## 6 CONCLUSION

This cross‐sectional descriptive study was conducted in a teaching hospital in SA to assess nurses’ perceptions of the risk factors for falls and fall prevention strategies. Nurses working in acute care settings must understand the intrinsic and extrinsic factors in patient falls and be aware of strategies to mitigate those risks. A routine and formal patient safety training once a year is one approach that could help nurses identify effective fall prevention interventions. The more effective the fall prevention interventions are, the greater the likelihood patients will be kept safe.

## CONFLICT OF INTEREST

The authors have no conflicts of interest to declare.

## ETHICS STATEMENT

The Institutional review board approval was obtained from King Saud University [E‐20–4850] prior to collecting data from participants. The study was performed following the Declaration of Helsinki.

## Supporting information

Supplementary Material

## Data Availability

The data that support the findings of this study are available from the corresponding author upon reasonable request.
